# Alteration of Anticancer and Protein-Binding Properties of Gold(I) Alkynyl by Phenolic Schiff Bases Moieties

**DOI:** 10.3390/pharmaceutics13040461

**Published:** 2021-03-29

**Authors:** Bandar A. Babgi, Jalal Alsayari, Hana M. Alenezi, Magda H. Abdellatif, Naser E. Eltayeb, Abdul-Hamid M. Emwas, Mariusz Jaremko, Mostafa A. Hussien

**Affiliations:** 1 Department of Chemistry, Faculty of Science, King Abdulaziz University, P.O. Box 80203, Jeddah 21589, Saudi Arabia; jalal.alsayari@gmail.com (J.A.); hana.tayma@hotmail.com (H.M.A.); mostafa_aly2@yahoo.com (M.A.H.); 2 Chemistry Department, Deanship of Scientific Research, College of Sciences, Taif University, Al-Haweiah, P.O. Box 11099, Taif 21944, Saudi Arabia; m.hasan@tu.edu.sa; 3 Department of Chemistry, College of Science and Arts, King Abdulaziz University, P.O. Box 344, Rabigh 21911, Saudi Arabia; nasertaha90@gmail.com; 4 Core Labs, King Abdullah University of Science and Technology (KAUST), P.O. Box 4700, Thuwal 23955-6900, Saudi Arabia; abdelhamid.emwas@kaust.edu.sa; 5 Biological and Environmental Science and Engineering (BESE), King Abdullah University of Science and Technology (KAUST), P.O. Box 4700, Thuwal 23955-6900, Saudi Arabia; 6 Department of Chemistry, Faculty of Science, Port Said University, Port Said 42521, Egypt

**Keywords:** gold(I), phosphine, HSA-binding, molecular docking, anticancer properties

## Abstract

A set of five gold complexes with the general formula Au(PR_3_)(C≡C-C_6_H_4_-4-R′) (R = PPh_3_, R′ = –CHO (1), R = PCy_3_, R′ = –CHO (2), R = PPh_3_, R′ = –N=CH-C_6_H_4_-2-OH (3), R = PPh_3_, R′ = –N=CH-C_6_H_4_-4-OH (4), R = PCy_3_, R′ = –N=CH-C_6_H_4_-2-OH (5)) were synthesized and characterized by elemental analysis, ^1^H-NMR spectroscopy, ^31^P-NMR spectroscopy, and mass spectrometry. The structures of complexes 2 and 5 were determined by X-ray crystallography. The effects of the structural modifications on the protein binding affinities and anticancer activities of the five gold complexes were assessed. Fluorescence quenching experiments to assess binding to human serum albumin (HSA) revealed that the Schiff base complexes (3, 4, and 5) had binding constants that were superior to their parent aldehyde complexes and highlighted the position of the hydroxy group because complex 4 (4-hydroxy) had a binding constant 6400 times higher than complex 3 (2-hydroxy). The anticancer activities of the complexes against the OVCAR-3 (ovarian carcinoma) and HOP-62 (non-small-cell lung) cancer cell lines showed that the Schiff bases (3–5) were more cytotoxic than the aldehyde-containing complexes (1 and 2). Notably, compound 4 had cytotoxic activity comparable to that of cisplatin against OVCAR-3, demonstrating the significance of the para position for the hydroxy group. Molecular docking studies against the enzyme thioredoxin reductase (TrxR) and human serum albumin were conducted, with docking scores in good agreement with the experimental data. The current study highlights how small structural modifications can alter physiochemical and anticancer properties. Moreover, this simple design strategy using the aldehyde group can generate extensive opportunities to explore new gold(I)-based anticancer drugs via condensation, cyclization, or nucleophilic addition reactions of the aldehyde.

## 1. Introduction

**Figure 1:**
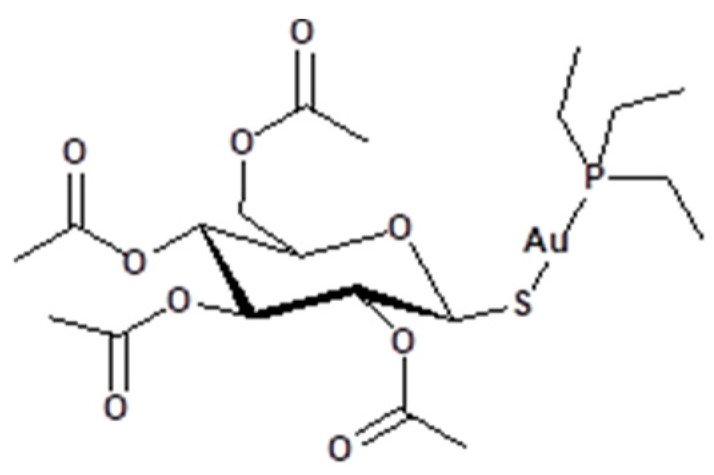
Auranofin is an anti-rheumatic agent with favorable anticancer activity.

Chemotherapy was established in the 1940s, and most of the early chemotherapeutic agents were organic. In 1978, the discovery of the anticancer activities of cisplatin expanded research into the metal complexes domain [1]. Since then, other platinum complexes analogous to cisplatin have been tested and approved clinically in the treatment of various types of cancers [2]. Despite the effiaciency of platinum-based chemotherapeutic agents, interest in other metal complexes has been growing in order to overcome some of the negative side effects of the platinum complexes [3]. In this context, gold(I)-based complexes have emerged as promising anticancer candidates [4,5,6,7,8,9]. Several gold(I) complexes have been examined for anti-tumor activity in vitro and in vivo [10,11,12]. In particular, numerous auranofin analogues with variations in the phosphine and thiolate ligands have been screened (Figure 1) [13]. The labile nature of the thiolate and their reducing capability has directed interest toward σ-bonded alkynyl ligands. Studies on complexes with a 4-ethynylanisole ligand and variant phosphine ligands revealed no strong differences in cytotoxicity (minimum cytotoxic effect of the phosphine ligands). Application of some of these complexes in vivo in mice was found to be challenging due to the low solubility of the complexes, which required a formulation strategy. Gold(I) complexes of the type Au(C≡CR)(PR_3_) have been reported to exhibit very strong inhibition of the enzyme thioredoxin reductase (TrxR) and showed high antiproliferative activity in tumor cells [14,15]. TrxR is a homo-dimeric protein classified as a glutathione reductase-like enzyme. It facilitates the NADPH-dependent reduction of thioredoxin (Trx) disulfide and many other oxidized cell constituents. The enzyme is involved in several processes, including protecting cells from oxidative stress, which is a major cause of DNA damage. The structural flexibilities of the alkynyl and phosphine ligands have allowed fine-tuning of physiochemical and pharmacological properties. Thus, different types of gold-based drugs have been reported in order to identify the appropriate ligand combinations with improved hydrophilic/lipophilic properties, cytotoxicity, and selectivity [16]. For example, the use of a water-soluble phosphine ligand (1,3,5-triaza-7-phosphaadamantane) improves the hydrophilic properties of gold complexes [8]. The strategy of our current work is to expand the structural flexibility of the alkynyl by employing the 4-ethynylbenzaldehyde with its very reactive aldehyde group. The aldehyde can undergo a range of condensation and cycloaddition reactions to generate well-known pharmacological scaffolds. The synthesis of Au(PR_3_)(C≡C-C_6_H_4_-4-CHO) was achieved, and the aldehyde-containing complexes were refluxed with aminophenols to generate Schiff bases. The complexes were then tested against two cancer cell lines, and their protein-binding activities to human serum albumin (HSA) were assessed. Molecular docking was undertaken to highlight the structural aspects of the complexes with HSA and thioredoxin reductase (TrxR). Molecular docking is a powerful tool in computer-assisted drug design with the goal to estimate the major binding modes of the drug with the 3D structure of the protein. Docking can be used to rationalize experimental findings, suggest structural–property correlations, and suggest inhabitation sites of the target, which are important for planning further optimization [17].

## 2. Materials and Methods

### 2.1. Materials

All solvents were obtained commercially and dried over molecular sieves (A4) before use. The term “petrol” refers to a fraction of petroleum ether with a boiling range of 40–60 °C. Chromatography was carried out on silica gel 60 particle sizes 0.063–0.200 mm (70–mesh ASTM) or basic ungraded alumina. Potassium tert-butoxide, p-aminophenol, and o-aminophenol were purchased commercially and used as received. 4-Ethynylbenzaldehyde [18], AuCl(PCy_3_) [19], AuCl(PPh_3_) [20], and Au(C≡C-C_6_H_4_-4-CHO)(PPh_3_) (1) [21] were prepared according to published procedures.

### 2.2. Methods and Instrumentation

**Figure 2:**
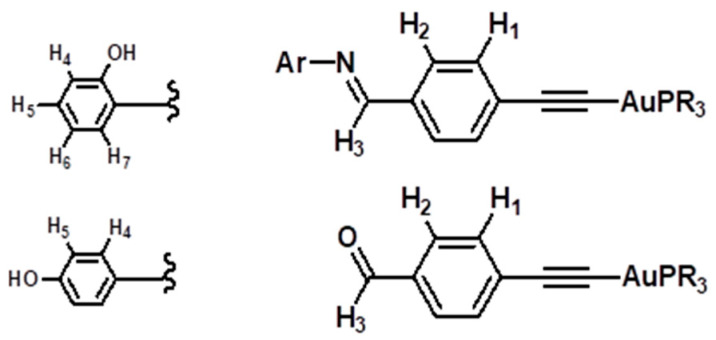
Numbering Scheme for NMR Spectral Assignment.

All reactions were carried out using standard Schlenk techniques, under a nitrogen atmosphere, unless otherwise stated. Infrared (IR) spectra were recorded using solid samples on a Perkin Elmer Spectrum 100 instrument (Shelton, CT, USA); peaks are reported in cm^−1^. Steady state emission spectra were recorded using Shimadzu RF 5301 PC spectrofluorometer (Columbia, Maryland, USA) using a rectangular quartz cell. High-resolution electrospray ionization (ESI) mass spectra were recorded using an Agilent Q-TOF 6520 instrument (Santa Clara, CA, USA); all mass spectrometry data are reported as m/z. ^1^H NMR (600 MHz) and ^31^P NMR (242 MHz) spectra were collected in CDCl_3_ using a Bruker Avance 600 MHz spectrometer furnished with a BBO probe (BrukerBioSpin, Rheinstetten, Germany); atom labeling follows the numbering in Figure 2.

### 2.3. Synthesis and Characterizations

Synthesis of OHC-4-C_6_H_4_-C≡C-Au-PCy_3_ (2): AuCl(PCy3) (0.357 g, 0.696 mmol) and Bu*^t^*OK (0.592 g, 5.281 mmol) were added to a solution of 4-ethynylbenzaldehyde (0.121 g, 0.931 mmol) in 25 mL methanol and 10 mL chloroform under N_2_ atmosphere, and the mixture was incubated overnight with stirring. The solvent was reduced under pressure and aqueous methanol (70%) was added, producing a yellowish precipitation. The precipitation was collected by filtration, affording 2 as a pale-yellow powder (0.347, 61%). HR ESI MS [C_21_H_38_AuP]: Calcd. 518.2377, found 518.2247. Elemental analysis for C_27_H_44_AuOP, Calcd (found): C, 53.47 (53.21) and H, 6.31 (6.07)%. IR (solid): 2108 cm^−1^ ν (C≡C), 1691 cm^−1^ ν (C=O). 1H NMR (CDCl_3_): δ 10.01 (s, 1H, H_3_), 7.82 (d, 2H, J_HH_ = 9 Hz H_2_), 7.60 (d, 2H, J_HH_ = 9 Hz, H_1_), 1.7–2.10 (m, 33H, PCy_3_). ^31^P NMR: δ 56.3 (s, PCy_3_).

Synthesis of HO–2–C_6_H_4_–N=C–4-C_6_H_4_-C≡C–Au-PPh_3_ (3): *O*-aminophenol (0.022 g, 0.202 mmol) and MgSO_4_ (0.279 g, 2.321 mmol) were stirred with a solution of compound 1 (0.099 g, 0.168 mmol) in a mixture of 20 mL methanol and 10 mL chloroform under N_2_ atmosphere at reflux for 5 h. The reaction mixture was evaporated to dryness, and 20 mL CHCl_3_ was added. The mixture was filtered and the filtrate was reduced to about 10 mL. A 30 mL portion of methanol was added, leading to a precipitation. The precipitation was collected by filtration and dried, offering 3 as a pale-yellow powder (0.050 g, 43%). HR ESI MS [C_33_H_26_AuNOP]: Calcd. 680.1412, found 680.1415. Elemental analysis for C_33_H_25_AuNOP, Calcd (found): C, 58.33 (57.91); H, 3.71 (3.36) and N, 2.06 (1.76)%. IR (solid): 3375 cm^−1^ ν (O–H), 2113 cm^−1^ ν (C≡C), 1621 cm^−1^ ν (C=N). ^1^H NMR (CDCl_3_): δ 8.87 (s, 1H, OH), 8.59 (s, 1H, H_3_), 7.86 (d, 2H, J_HH_ = 9 Hz, H_2_), 7.57–7.42 (m, 15H, PPh_3_), 7.35 (d, 2H, J_HH_ = 9 Hz, H_1_), 7.12 (d, 1H, J_HH_ = 8 Hz, H_7_), 6.99 and 6.74 (2 t, 2H, J_HH_ = 8 Hz, H_4_ and H_6_), 6.80 (d, 1H, J_HH_ = 8 Hz, H_5_). ^31^P NMR: δ 41.6 (s, PPh_3_).

Synthesis of HO–4–C_6_H_4_–N=C–4-C_6_H_4_-C≡C–Au-PPh_3_ (4): *P*-aminophenol (0.024 g, 0.220 mmol) and MgSO_4_ (0.182 g, 1.513 mmol) were stirred with a solution of compound 1 (0.126 g, 0.214 mmol) in a mixture of 20 mL methanol and 10 mL chloroform under N_2_ atmosphere at reflux for 5 h. The reaction mixture was evaporated to dryness and 20 mL CHCl_3_ was added. The mixture was filtered and the filtrate was reduced to about 10 mL. A 30 mL portion of methanol was added, leading to a precipitation. The precipitation was collected by filtration and dried, offering 4 as a pale-yellow powder (0.140 g, 96%). HR ESI MS [C_33_H_26_AuNOP]: Calcd. 680.1412, found 680.1415. Elemental analysis for C_33_H_25_AuNOP, Calcd (found): C, 58.33 (58.04); H, 3.71 (3.49) and N, 2.06 (1.82)%. IR (solid): 3360 cm^−1^ ν (O-H), 2140 cm^−1^ ν (C≡C), 1620 cm^−1^ ν (C=N). ^1^H NMR (CDCl_3_): δ 8.33 (s, 1H, H_3_), 7.69 (d, 2H, J_HH_ = 9 Hz, H_2_), 7.49 (d, 2H, J_HH_ = 9 Hz, H_1_), 7.44–7.36 (m, 16H, OH and PPh_3_), 7.05 (d, 2H, J_HH_ = 9 Hz, H_4_), 6.60 (d, 2H, J_HH_ =9 Hz, H_5_). ^31^P NMR: δ 42.12 (s, PPh_3_).

Synthesis of HO–2–C_6_H_4_–N=C–4-C_6_H_4_-C≡C–Au-PCy_3_ (5): *O*-aminophenol (0.082 g, 0.752 mmol) and MgSO_4_ (0.419 g, 3.481 mmol) were stirred with a solution of compound 2 (0.302 g, 0.501 mmol) in a mixture of 20 mL methanol and 10 mL chloroform under N_2_ atmosphere at reflux for 5 h. The reaction mixture was evaporated to dryness and 20 mL CHCl_3_ was added. The mixture was filtered and the filtrate was reduced to about 10 mL. A 30 mL portion of methanol was added, leading to a precipitation. The precipitation was collected by filtration and dried, offering 5 as a pale-yellow powder (0.302 g, 57%). HR ESI MS [C_33_H_44_AuNOP]: Calcd. 698.2829, found 698.2822. Elemental analysis for C_33_H_43_AuNOP, Calcd (found): C, 56.81 (56.48); H, 6.21 (5.87) and N, 2.01 (1.64)%. IR (solid): 3411 cm^−1^ ν (O–H), 2108 cm^−1^ ν (C≡C), 1622 cm^−1^ ν (C=N). ^1^H NMR (CDCl_3_): δ 8.64 (s, 1H, H_3_), 7.79 (d, 2H, J_HH_ = 9 Hz, H_2_), 7.58 (d, 2H, J_HH_ = 9 Hz, H_1_), 7.29 (1H, J_HH_ = 9 Hz, H_7_), 7.17 and 6.89 (2 t, 2H, J_HH_ = 9 Hz, H_4_ and H_6_), 6.99 (d, 1H, H_5_), 1.7–2.10 (m, 33H, PCy_3_). ^31^P NMR: δ 56.30 (s, PCy_3_).

### 2.4. Crystal Structure Determination

The sample crystals of 2 and 5 were crystallized in a chloroform–methanol mixture under slow evaporation. Crystals were mounted on Bruker D8 Quest Diffractometer, equipped with micro-focused Mo Kα radiation for data collection. Data were collected using APEX3 software (SAINT and SADBAS, 2016, Bruker AXS Inc., Madison, WI, USA) [22] at 296 K with Mo Kα radiation. The structure solution was performed using SHELXS–2014 [23] and refined by full-matrix least-squares methods on F2 using SHELXL–2014 [23]. All non-hydrogen atoms were refined anisotropically by full-matrix least-squares methods. The figures were generated through APEX3 software [22]. All hydrogen atoms were positioned geometrically and treated as riding atoms with C–H = 0.93 Å and Uiso(H) = 1.2 Ueq(C) for all carbon atoms. The crystal data were deposited at the Cambridge Crystallographic Data Centre with deposition numbers 2063561 and 2063564. Crystal data can be obtained free of charge from CCDC, 12 Union Road, Cambridge CB21 EZ, UK (Fax: (+44)-1223-336-033; e-mail: data_request@ccdc.cam.ac.uk).

Crystal data of complex 2: monoclinic; P1 21/c1; unit cell dimensions: a = 12.3170(7) Å, b = 16.5507(8) Å, c = 12.5410(6) Å, α = 90°, β = 97.451(2)°, γ = 90°; volume = 2535.0(2) Å^3^; absorption coefficient: 5.882 mm^−^^1^; Theta range for data collection: 2.50 to 25.35°; Reflections collected: 53717; Independent reflections: 4629 [R(int) = 0.0760].

Crystal data of complex 5: monoclinic; P1 21/n1; unit cell dimensions: a = 12.8647(11) Å, b = 16.2774(14) Å, c = 28.952(3) Å, α = 90°, β = 99.970(3)°, γ = 90°; volume = 5971.1(9) Å^3^; absorption coefficient: 5.007 mm^−1^; Theta range for data collection: 2.25 to 25.43°; Reflections collected: 157,459; Independent reflections: 11,000 [R(int) = 0.2339].

### 2.5. HSA Binding Studies

Human serum albumin (HSA) solution (32 µM) was prepared in Tris-HCl/NaCl aqueous buffer (pH = 7.4). The solution was titrated with different concentrations of complexes 1–5 in DMSO (the amount of DMSO was maintained at ca. 20% *v/v*). The changes in the emission intensity of the protein were followed at around 330 nm upon excitation at 275 nm after incubating the complexes for 4 min at 295 K. The Stern–Volmer quenching constants (K_SV_) were obtained from Equation (1), where I_o_ and I are the emission intensities of the protein in the absence and the presence of the complexes, respectively. The [Au complex] was plotted against [I_o_/I]; the K_SV_ value was defined as the slope of the line [24]. The Stern–Volmer constant is related to the quenching constant by Equation (2), where K_q_ is the quenching rate constant, and τ_0_ is the average lifetime of the fluorescence for free HSA (τ_0_ = 7 ns) [25,26]. The binding constant (K_b_, M^−1^) and the number of binding sites (n) were calculated by plotting log[I_o_ − I/I] against Log [Au complex], where the slope equals n and the intercept is equivalent to Log K_b_ according to Equation (3) [27]. Gibbs free energy (∆G^0^) of the binding to HSA protein can be calculated by employing Equation (4), using the binding constant (K_b_, M^−1^) [27,28].

I_0_/I = 1 + K_sv_ [Au complex] (1)

K_sv_ = K_q_ × τ_0_ (2)

Log(I_0_ − I)/I = Log K_b_ + n Log [Au complex] (3)

∆G^0^ = −R × T × Ln × K_b_ (4)

### 2.6. Molecular Docking Studies

The structures of the human thioredoxin reductase “hTrxR1” receptor, which is a prominent anticancer drug target [29], and the human serum albumin “HSA” receptor, which is used as an anticancer drug carrier [30], were obtained from the protein data bank (PDB: 3QFB and 1H9Z) (http://www.rcsb.org/pdb/home/home.do “10 June 2020”) [31,32]. The gold complexes were drawn in ChemBioOffice ultra version 13. All docking studies were operated using the MOE program; all water and cofactor molecules were removed from the downloaded Human hTrxR1 thioredoxin reductase “3QFB” and HSA ”1H9Z”. After that, all invalid charges and broken bonds were fixed, and all hydrogen atoms were added after the preparation. The parameters and charges were consigned with MMFF94x force field. After alpha-site spheres were identified using the site finder module of MOE, the compounds were docked to the same active site of the co-crystalized compound using the DOCK module of MOE [33,34]. The docking scores in the MOE software were collected utilizing the London dG scoring function. The highest ten docking scores were used to compare between the five organometallic compounds and the co-crystalline reference compound; optimization of the scoring values were processed by two independent refinement methods. The docking results were validated following the reported pose selection method, which involves re-docking the co-crystalized ligand into the receptor’s active site [35,36], and confirmed the ability of the program to identify the best pose under a preselected root mean square deviation (RMSD) value from the known conformation (regularly 1.5 or 2 Å depending on ligand size). In the current study, pose selection and docking score for flavin-adenine dinucleotide (the co-crystalized compound with 3QFB) and R-warfarin “(the co-crystalized compound with 1H9Z)” were determined; the docking result of the same compound reached a 1.09 Å and 1.82 resolution of the co-crystalline structures.

### 2.7. Anticancer Studies

The cells were obtained by the Egyptian Holding Company for Biological Products and Vaccines (VACSERA) and then maintained in the tissue culture unit. Cells were grown in RPMI-1640 medium, supplemented with 10% heat inactivated FBS, 50 units/mL of penicillin, and 50 mg/mL of streptomycin, and maintained in a humidified atmosphere with 5% carbon dioxide [37,38]. The cells were kept as monolayer cultures by serial sub-culturing, using cell culture reagents from Lonza (Basel, Switzerland). The antitumor activities of the complexes were assessed against OVCAR-3 (ovarian carcinoma cancer) and HOP-62 (non-small-cell lung cancer) cell lines.

The sulforhodamine B (SRB) assay method was applied to determine the cytotoxicity, as described in the literature [39]. Exponentially growing cells were collected using 0.25% Trypsin-EDTA and seeded in 96-well plates at 1000–2000 cells/well in RBMI-1640 supplemented medium. The cells were kept in the medium for 24 h and then incubated for 3 days with various concentrations of the gold compounds. Following 3 days of treatment, the cells were fixed with 10% Cl_3_CCOOH for 60 min at 4 °C. Wells were stained for 10 min at room temperature with 0.4% SRBC dissolved in 1% CH_3_COOH. The plates were air dried for 24 h, and the dye was dissolved in Tris-HCl for 5 min with shaking at 1600 rpm. The optical density of each well was evaluated spectrophotometrically at 564 nm with an ELISA microplate reader (ChroMate-4300, Orlando, FL, USA). The half minimal inhibitory concentration (IC50) values were calculated from a Boltzmann sigmoidal concentration response curve using nonlinear regression fitting models (Graph Pad, Prism Version 9, San Diego, CA, USA).

## 3. Results and Discussion

### 3.1. Synthesis and Characterization

**Figure 3:**
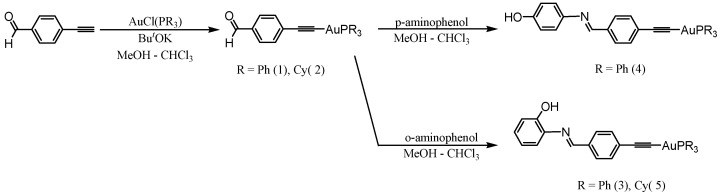
Synthesis of the gold(I) alkynyl containing Schiff bases.

**Figure 4:**
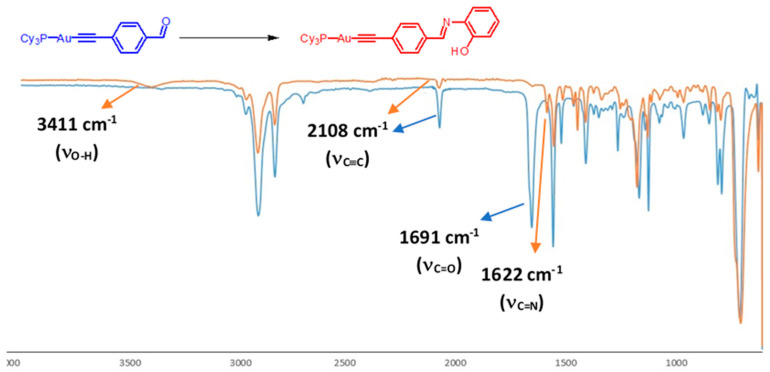
The changes in the IR spectra upon reacting 2 with 2-aminophenol to produce 5.

Phenolic groups possess several properties: the possibility to form highly water-soluble sodium phenoxide salts, the weak acid nature in pH > 8 that allows the formation of phenoxide anions in solutions, and the possibility to form hydrogen bonding. These features are attractive in drug design, and hence phenolic moieties were chosen to be incorporated into the gold complexes. The route was established by synthesizing complexes (1) and (2) by reacting 4-ethynylbenzaldehyde with AuCl(PR_3_) in the presence of an excess amount of potassium tert-butoxide in a methanol/chloroform mixture [40]. The ^31^P NMR spectra of (1) and (2) exhibited one singlet around 40 and 56 ppm, respectively. The absence of the (≡C–H) band at 3200 cm^−1^ in the IR spectra indicates that gold-bonded C≡C units are present. The Schiff bases (3), (4), and (5) were obtained by reaction of excess amounts of o-aminophenol or p-aminophenol with the gold(I) alkynyl aldehyde in refluxing chloroform and methanol for 5 h in the presence of anhydrous magnesium sulfate as a drying agent. The reaction mixture was filtered, and the solvent was removed to produce the desired product (Figure 3). The ^1^H NMR spectra of the Schiff bases showed sharp singlets of the azomethine (–CH=N–) in the range 8.3–8.9 ppm associated with the disappearance of the aldehyde proton signal around 10.0 ppm (All the NMR data are provided in the supplementary materials Figures S1–S8). The FTIR spectra of the gold alkynyl Schiff bases showed intense bands around 1620 cm^−1^ for the azomethine bond –CH=N; no bands around the 1700 cm^−1^ region were observed, confirming that the aldehyde groups are converted into –CH=N groups. Additionally, bands in the range 3100–3500 cm^−1^ for stretching OH bond were observed for the complexes 3–5 (Figure 4).

### 3.2. Crystallographic Studies

**Figure 5:**
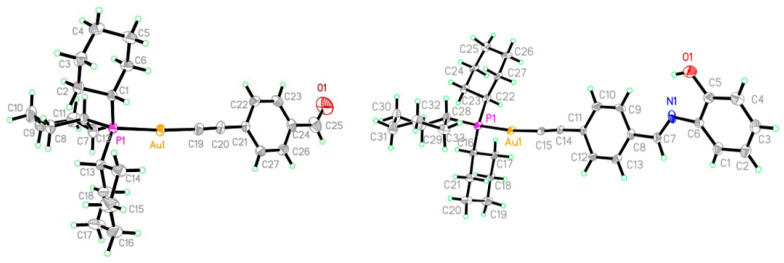
Crystal structures of 2 (**left**) and 5 (**right**).

X-ray diffraction studies were completed for complexes 2 and 5. The molecular structures with the atom-numbering scheme is given in Figure 5. Crystallographic data are listed in Section 2.4, while important bond lengths are provided in Table 1. The structural determinations confirm the molecular composition extracted from the spectral data. The Au1-P1 distances are 2.293(1) Å in complex 2 and 2.293(2) Å [2.293(2)] in complex 5. The P-Au-C angles are 175.81(14)° and 174.4(2)° (177.9(3)°) for complexes 2 and 5, respectively, while Au-C≡C angles are 177.6(5)° and 176.1(8)° [176.7(8)°] for complexes 2 and 5, respectively, which show a slight deviation from linearity. In complex 5, the azomethine bond, N1-C7, is 1.237(12) Å (1.267(12) Å) and is in conformity with a formal C=N double bond, and the C5-O1 bond distance of 1.379(12) Å (1.368(16) Å) is slightly shorter than the normal C–O single-bond distance. The bond distance for C6-N1 is 1.419(13) Å (1.400(14) Å), which is close to the C–N single bond. This compound exits in the imine-ol form clearly.

### 3.3. HSA Binding

**Figure 6:**
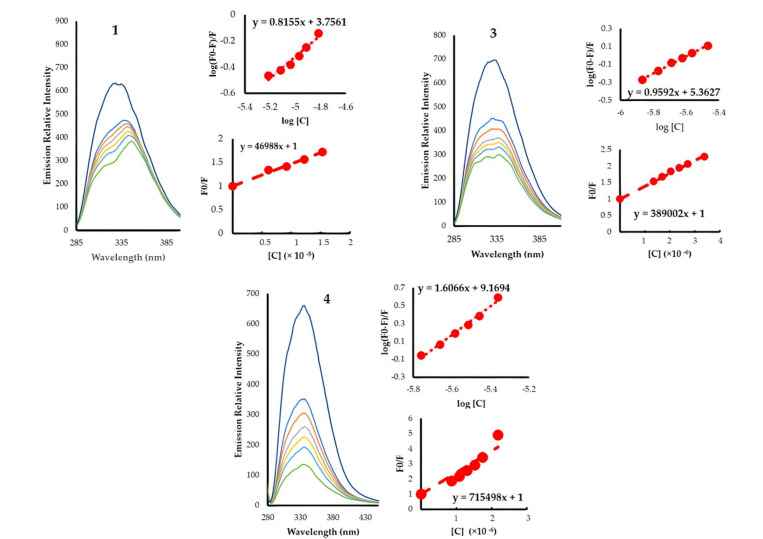
Binding studies of 1, 3 and 4 with HSA.

The interaction of chemotherapeutic drugs with blood plasma proteins has been under intensive investigation in recent years due to their function in drug transport and metabolism, principally with serum albumin, which accounts for 55% of the protein in blood plasma [41,42]. The interaction of the complexes with HSA were assessed by HSA fluorescence quenching (at 335–340 nm) upon increasing the concentration of the complexes at 295 K (Figure 6). The characteristic emission originating from the tryptophan units in HSA diminishes significantly with minute additions of the complexes, confirming the interaction process between the gold complexes and HSA. The data for the five complexes are summarized in Table 2. The quenching process can be due to the collisions between the excited fluorophore (HSA) with the quencher (dynamic quenching) or to the interaction between the fluorophore and the quencher at the ground state forming a non-fluorescent complex (static quenching). The quenching constants indicate a static quenching mechanism, as our complexes have rates much higher than 2.00 × 1010 M s^−1^ (maximum rate for dynamic quenching) [43]. From Equation (3) (Scatchard equation), the binding constants were extracted, highlighting the importance of the structural modifications on the binding to HSA (Table 2). Replacing triphenylphosphine with tricyclohexylphosphine, as in proceeding from 1 to 2 and 3 to 5, caused a 30- to 170-fold enhancement in the binding affinity as a result of increasing the hydrophobicity. Introducing the o-phenolic moiety, as in proceeding from 1 to 3 and 2 to 5, also induced strong increases in the binding affinities (40- and 240-fold increases, respectively). HSA is composed of three domains (DI, DII, and DIII), two of which are hydrophobic regions [42,44,45]. The effect induced by the phenolic group is believed to be produced by a combination of an increase in the hydrophobicity as well as the capability of the hydroxy group to establish hydrogen-bonding and dipole interactions. The role of the hydroxy group can be realized by comparing the effect of an o-phenolic against a p-phenolic moiety; the latter has binding affinities 6400 times higher due to the absence of intramolecular hydrogen bonding with the azomethine and the less steric demanding position for interactions. All the gold complexes exhibited negative values of ΔG^0^, demonstrating the spontaneous interaction with HSA protein with a trend similar to that observed in the binding affinities (Table 2). The average binding site count for our complexes followed the same trend seen in Kb; however, complex 4 (with the para hydroxy) is the only one that shows “n” that approaches 2. Indeed, the number of binding sites is more accurately describing the stoichiometric ratio of the compounds to HSA [46], and hence complex 4 could be able to interact with HSA at two different sites.

### 3.4. Anticancer Studies

The anticancer properties of complexes 1–5 were assessed against ovarian carcinoma (OVCAR-3) and human lung adenocarcinoma (HOP-62) cell lines (see Table 3). The obtained IC50 values for complexes 1 and 2 (the aldehyde containing complexes) ranged from 12.45 to 15.86 µM. However, the Schiff base complexes 3–5 exhibited much higher anticancer activities, with values ranging between 5.27 and 9.40 µM. Indeed, the IC50 values for the Schiff base-containing complexes are within a narrow range, which does not allow the extraction of conclusive structure–activity relationships; however, complex 4 seems to be more cytotoxic against OVCAR-3 when compared to the other complexes, indicating a possible preference for the para position for the hydroxy group.

### 3.5. Molecular Docking

**Figure 7:**
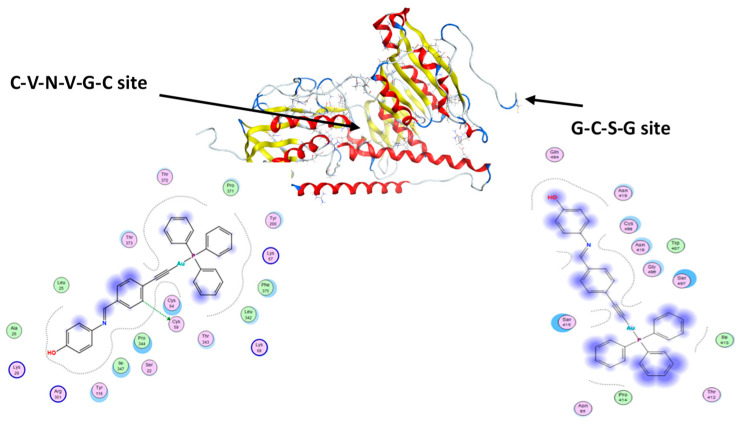
The interactions of complex 4 in the two active sites of the TrxR enzyme.

Auranofin and other gold(I) complexes have been reported to selectively inhibit the enzyme TrxR, which is regarded as the main target of gold complexes [47]. Overexpression of TrxR has been observed in numerous cancers and tumor cell lines, and cisplatin is one of the most effective inhibitors of TrxR [48]. The active sites of TrxR contain the amino acids selenocysteine (Sec) and cysteine (Cys), which contribute to the mechanism of action of the enzyme [48]. Gold complexes such as auranofin are believed to interact with the nucleophilic sulfur and selenium as their inhibitory mode of action. Thus, we selected this receptor from the PDB for our docking studies, also given recent studies on its role in cancer [49,50,51]. Molecular docking studies were conducted for our complexes in the two active sites of the human TrxR enzyme (Figure 7), using auranofin as a benchmark for comparison (Table 4). Auranofin has a greater preference for binding to site 1. Complexes 1, 3, 4, and 5 had docking scores similar to auranofin or higher in site 1. However, the aldehyde-containing complexes (1 and 2) bound more strongly to site 2. Introducing the Schiff base phenolic moieties altered the binding sites of the gold complexes, directing them toward site 1, which may be related to their greater cytotoxic effects when compared to their aldehyde precursors. Apart from van der Waals and hydrophobic interactions, complex 4 is the only complex that forms a hydrogen-donor interaction with cystine (Cys 59) in site 1, rationalizing the greater anticancer activity when compared to the other complexes. It is worth mentioning that auranofin is the only complex able to form similar interactions (H-donor with cystine) but in site 2.

**Figure 8:**
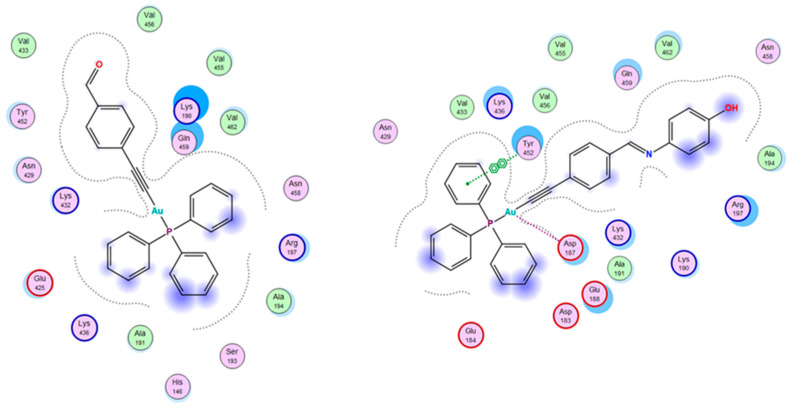
Comparison between the interactions of complexes 1 and 4 in the binding sites of HSA as predicted by MOE.

We next undertook an additional molecular docking study to gain further insight into the binding sites and possible interactions of the complexes to another protein, human serum albumin (HSA) [52]. HSA was chosen because of its high levels in human serum and its potential value as a ligand carrier for targeted anticancer drug delivery. The Schiff base complexes had better binding scores when compared to their parent aldehyde complexes. The obtained docking scores were in good agreement with the experimental data; hence, molecular docking can be used to shed some light on the structural aspects of the binding between the complexes and protein. The data indicated that all the complexes have a strong preference to bind to the DII domain. The HSA pocket (DII) is surrounded by Asp183, Glu184, Asp187, Glu188, Lys190, Ala191, Ala194, Arg197, Asn429, Lys432, Val433, Lys436, Tyr452, Val455, Val456, Asn458, Gln459, and Val462 amino acids; some of these amino acid residues are involved in the hydrophobic and van der Waals interactions with the complexes; 3 and 4 in particular are involved in a π–π interaction with Try452 (Figure 8). Complex 2 is positioned slightly differently in the same pocket.

## 4. Conclusions

In the current work, a simple strategy is introduced to structurally alter the anticancer activities of gold complexes with the general formula Au(PR3)(C≡C-Ar). *P*-ethynylbenzaldyde was used as a building block due to the reactive nature of the aldehyde group. We chose to pursue this strategy via reacting the aldehyde containing complexes with 2-aminophenol and 4-aminophenol, forming Schiff base gold complexes 3–5. The effect of the structural modifications was assessed by studying the protein binding affinities of the gold complexes as well as their cytotoxic effects on two cancer cell lines. The HSA-binding study was conducted by fluorescence quenching assay and showed that the Schiff base complexes had a much greater binding capacity than their parent aldehyde complexes; furthermore, the impact of the position of the hydroxy group was pronounced, as complex 4 (4-hydroxy) displayed a binding constant 6400 times higher than complex 3 (2-hydroxy). The IC50 of the complexes against OVCAR-3 and HOP-62 cell lines highlighted the functional significance of the structural modifications; the Schiff bases (3–5) generally had greater cytotoxic effects than the aldehyde-containing complexes (1 and 2). Moreover, compound 4 had cytotoxic activity comparable to that of cisplatin against OVCAR-3, suggesting the importance of the relative position of the hydroxy group. The structural–properties relationships were further explored by molecular docking studies against the enzyme thioredoxin reductase (TrxR), a well-known target for gold complexes in cancer cell lines, and human serum albumin. The docking scores were in agreement with the observed experimental data, highlighting how small structural modifications can alter physiochemical properties. The current synthetic strategy provides extensive possibilities for exploring new gold(I)-based anticancer drugs, as the aldehyde group can undergo a large range of condensation, cyclization, and nucleophilic addition reactions.

## Figures and Tables

**Table 1:** Bond lengths (Å) for complexes 2 and 5.

Complex 2		Complex 5	
Au1-C8	2.006(4)	Au1-C15	2.001(9)–2.007(9)
P1-C22	1.842(4)	P1-C22	1.832(9)–1.834(9)
O1-C9	1.175(7)	N1=C7	1.237(12)–1.267(12)
Au1-P1	2.2931(10)	Au1-P1	2.293(2)

**Table 2:** Kinetic and thermodynamic parameters of human serum albumin (HSA)-binding at 295 K.

Compound	K_sv_ (×10^4^)	K_q_ (×10^12^)	K_b_	n	∆G^0^ (kJ mole^−1^)
1	4.7	6.71	5.70 × 10^3^	0.82	−21.2
2	2.8	4.00	1.69 × 10^5^	1.16	−29.9
3	38.9	55.57	2.31 × 10^5^	0.96	−30.29
4	71.5	102.21	1.48 × 10^9^	1.61	−51.8
5	37.1	52.96	4.03 × 10^7^	1.37	−42.95

**Table 3:** Anticancer activities of the gold compounds and cisplatin in DMSO solutions.

Compound	OVCAR-3 (Ovarian Carcinoma Cancer Cell Line) (IC50% in µM)	HOP-62 (Non-Small-Cell Lung Cancer Cell Line) (IC50% in µM)
1	13.65 ± 0.53	12.45 ± 0.43
2	15.86 ± 0.53	14.82 ± 0.13
3	09.40 ± 0.17	07.25 ± 0.21
4	05.27 ± 0.11	08.16 ± 0.43
5	09.11 ± 0.12	07.55 ± 0.43
cisplatin	05.89 ± 0.12	03.91 ± 0.20

**Table 4:** Binding scores for Au-complexes as calculated by MOE.

Complexes	Docking Scores against Human Thioredoxin Reductase (TrxR) (3QFB)		Docking Scores against HSA (1H9Z)
	Site 1	Site 2	
1	−8.54	−8.69	−7.22
2	−7.79	−9.03	−6.98
3	−8.09	−8.13	−7.99
4	−8.30	−7.92	−8.21
5	−8.43	−7.65	−8.21
auranofin	−8.14	−7.65	-

## Data Availability

The data presented in this study are available on request from the authors.

## Supplementary Materials

The following are available online at https://www.mdpi.com/article/10.3390/pharmaceutics13040461/s1, Figures S1–S4: ^1^H NMR data, Figures S5–S8: ^31^P NMR data.

## Sample Availability

Not available.

## Abbreviations

HSA: Human serum albumin; OVCAR-3: Ovarian carcinoma cancer cell line; HOP-62: Non-small-cell lung cancer cell line.
