# Supplementary material for: Alteration of Anticancer and Protein-Binding Properties of Gold(I) Alkynyl by Phenolic Schiff Bases Moieties
Source: Pharmaceutics. 2021 Mar 29;13(4):461. doi: 10.3390/pharmaceutics13040461 (PMC8066730; doi:10.3390/pharmaceutics13040461)
Supplement: Supplementary file 1 [file pharmaceutics-13-00461-s001.pdf]

pharmaceutics

MDPI

Supplementary Materials: Alteration of Anticancer and Protein-binding Properties of Gold(I) Alkynyl by Phenolic Schiff Bases Moieties

Bandar A. Babgi *, Jalal Alsayari, Hana M. Alenezi, Magda H. Abdellatif, Naser E. Eltayeb, Abdul-Hamid M. Emwas, Mariusz Jaremko * and Mostafa A. Hussien

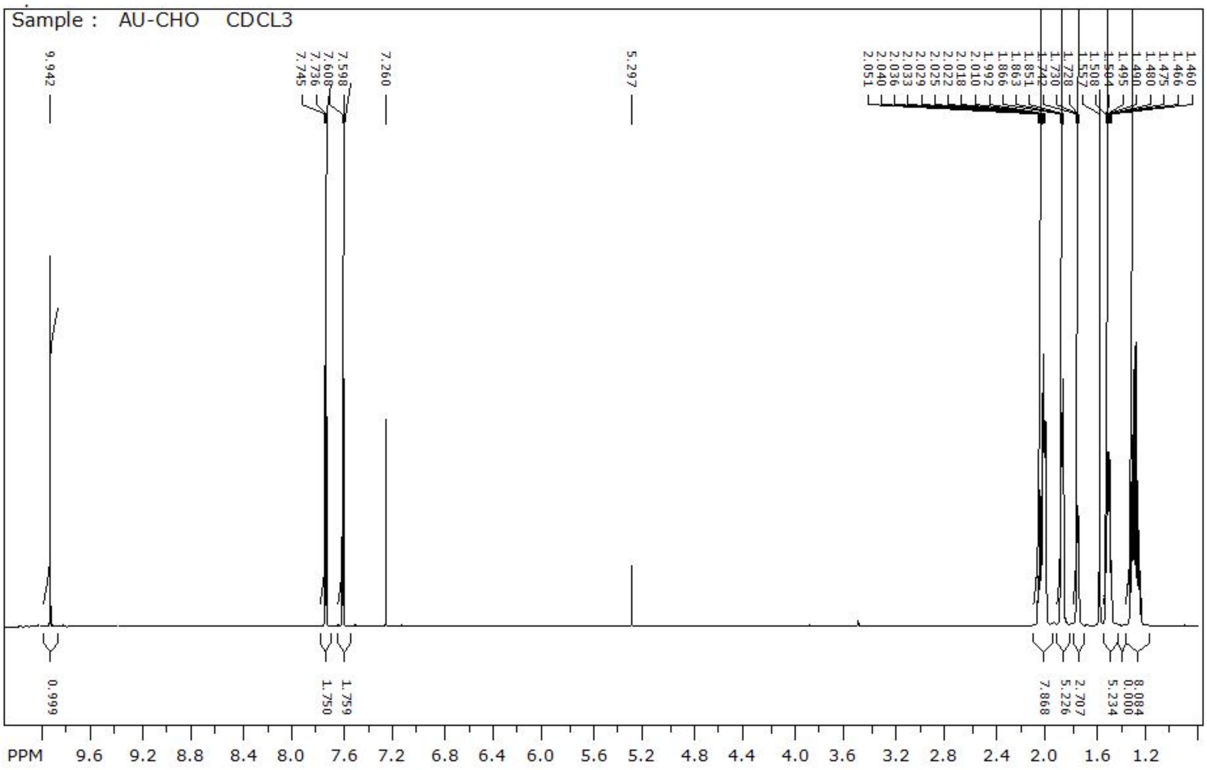

Figure S1. ^{1}H NMR for complex 2.

Publisher's Note: MDPI stays neutral with regard to jurisdictional claims in published maps and institutional affiliations.

Copyright: © 2021 by the authors. Submitted for possible open access publication under the terms and conditions of the Creative Commons Attribution (CC BY) license (http://creativecommons.org/licenses/by/4.0/).

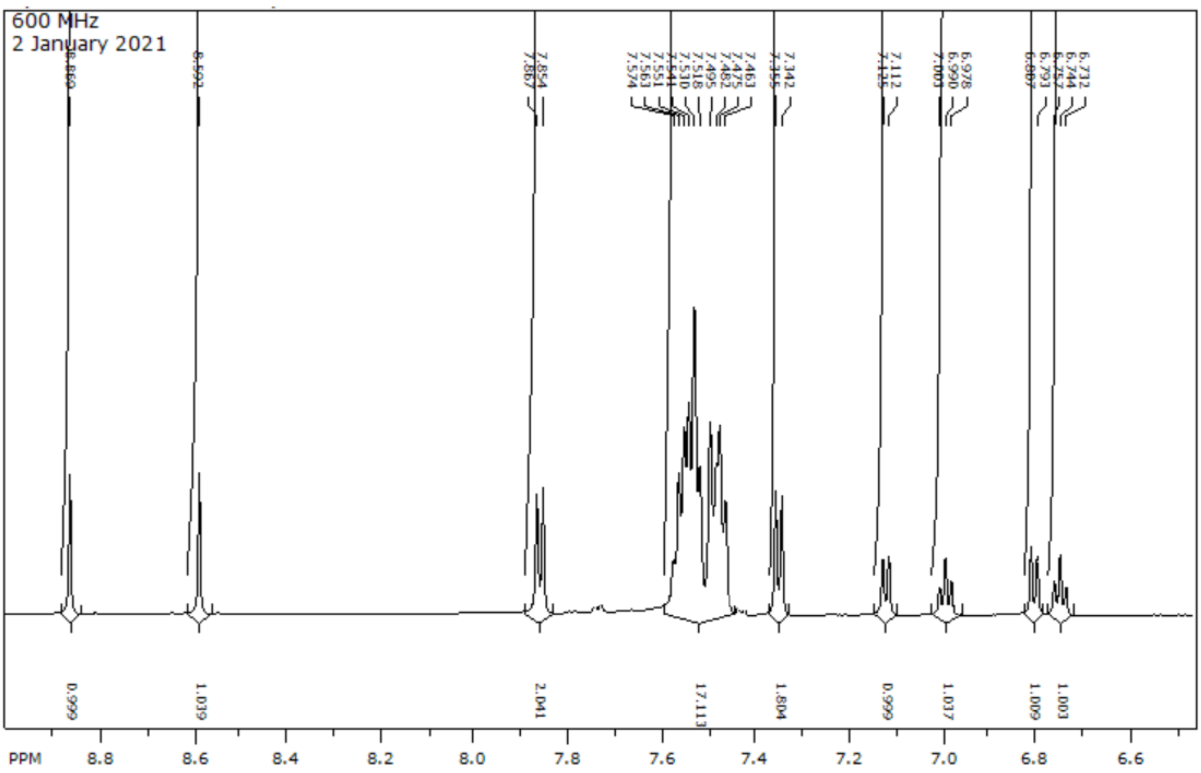

Figure S2. 1H NMR for complex 3.

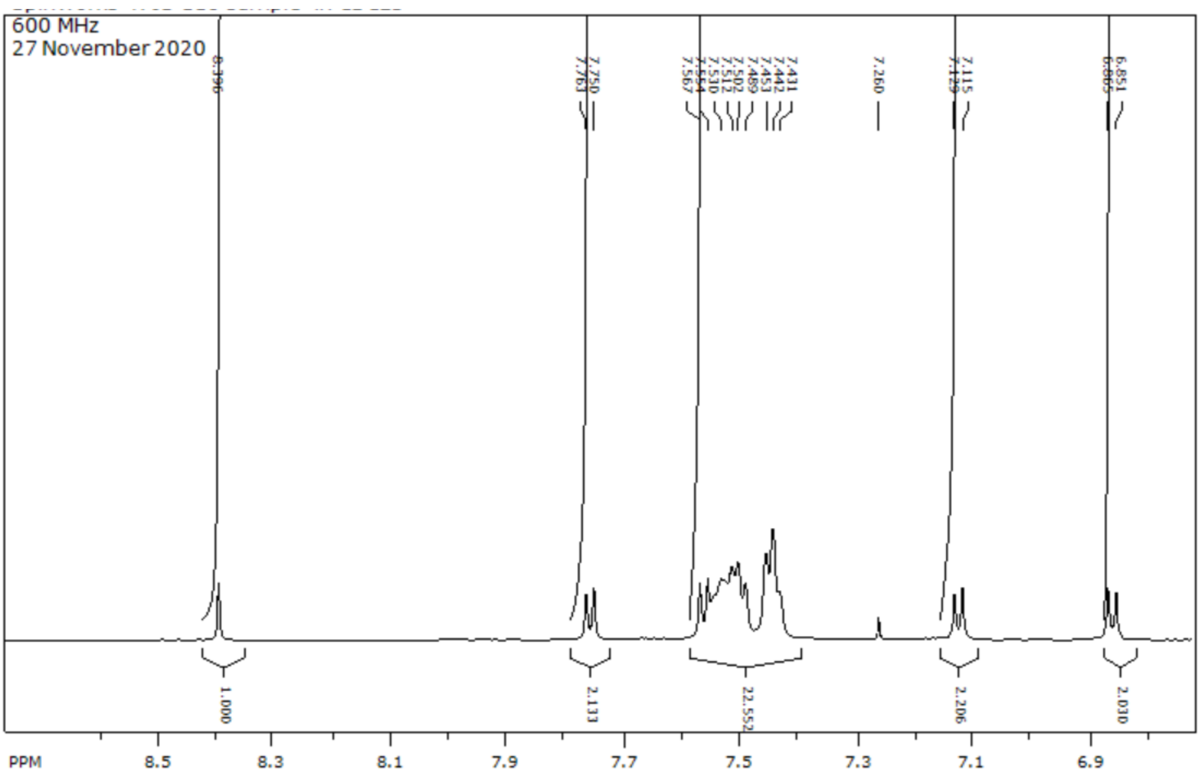

Figure S3. ^{1}H NMR for complex 4.

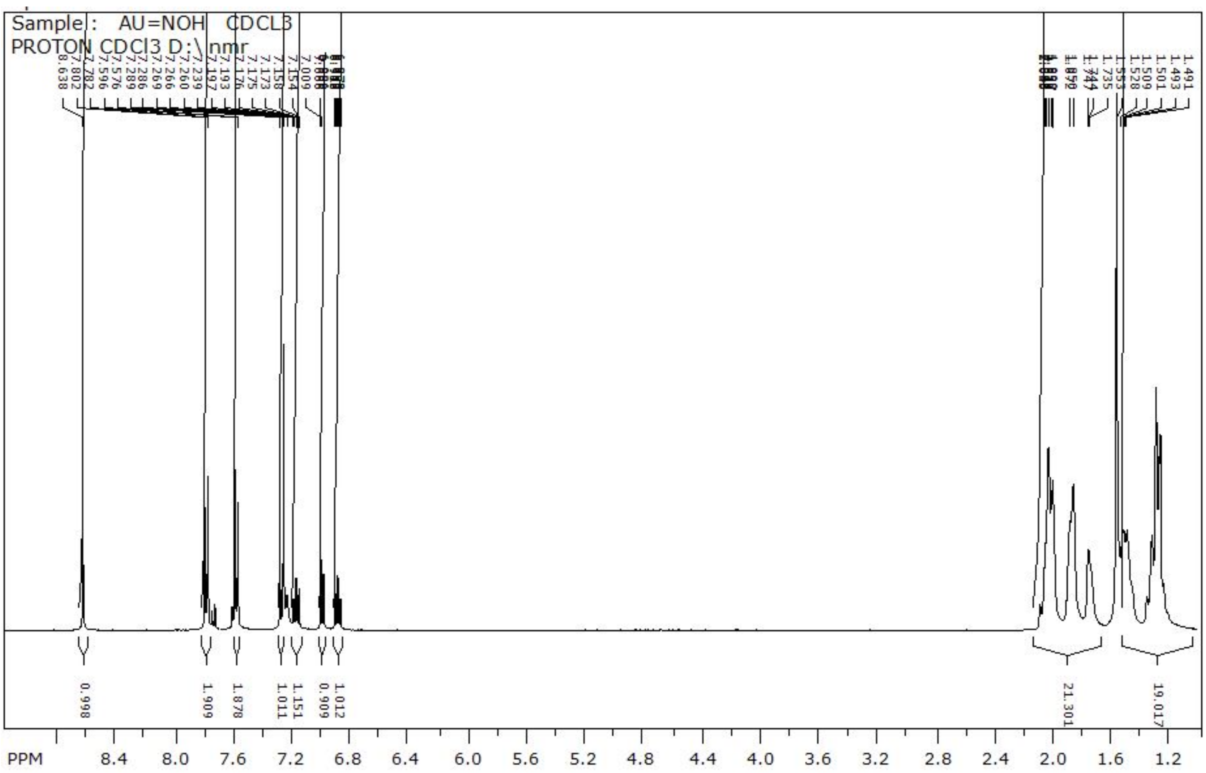

Figure S4. ^{1}H NMR for complex 5.

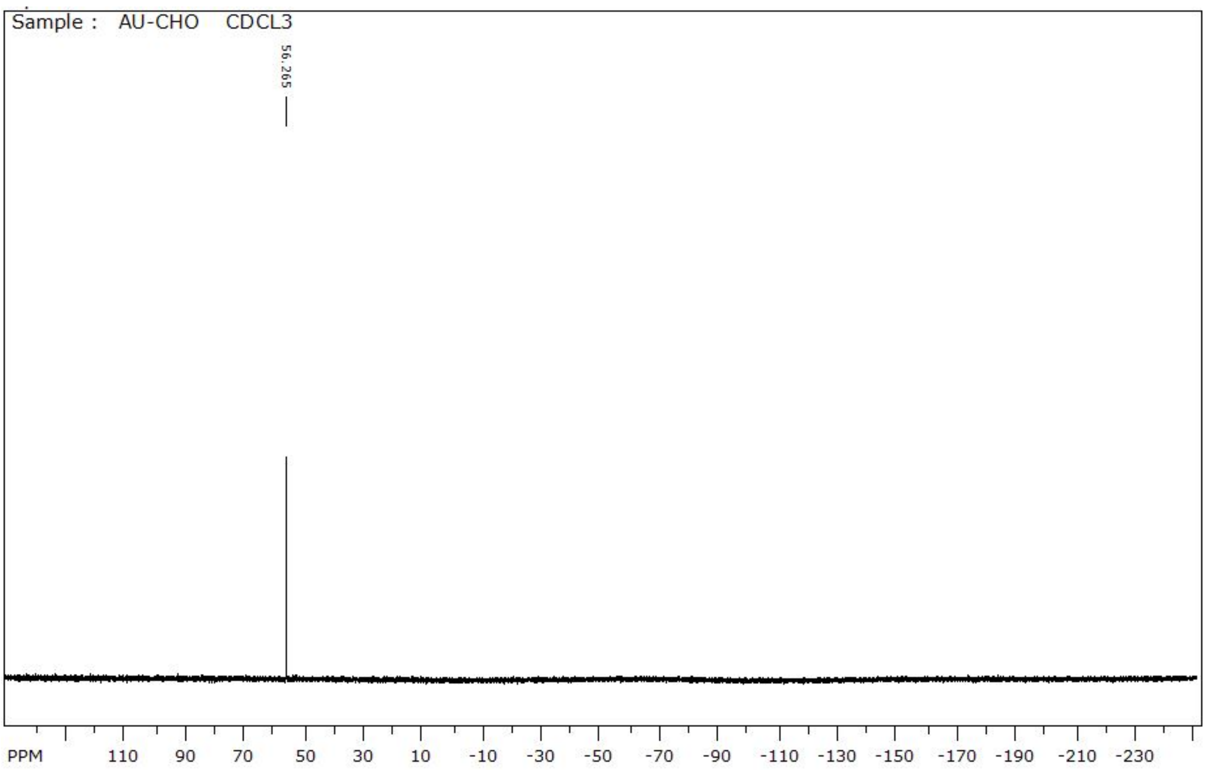

Figure S5. ^{31}P NMR for complex 2.

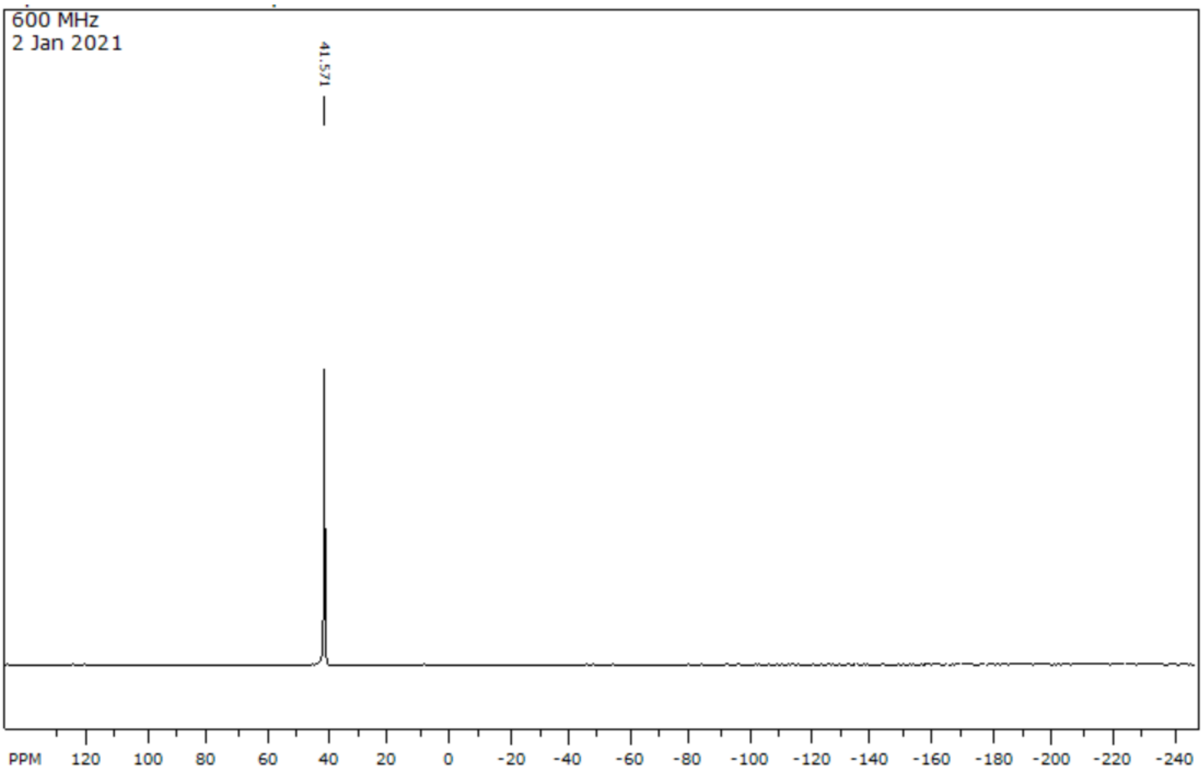

Figure S6. ^{31}P NMR for complex 3.

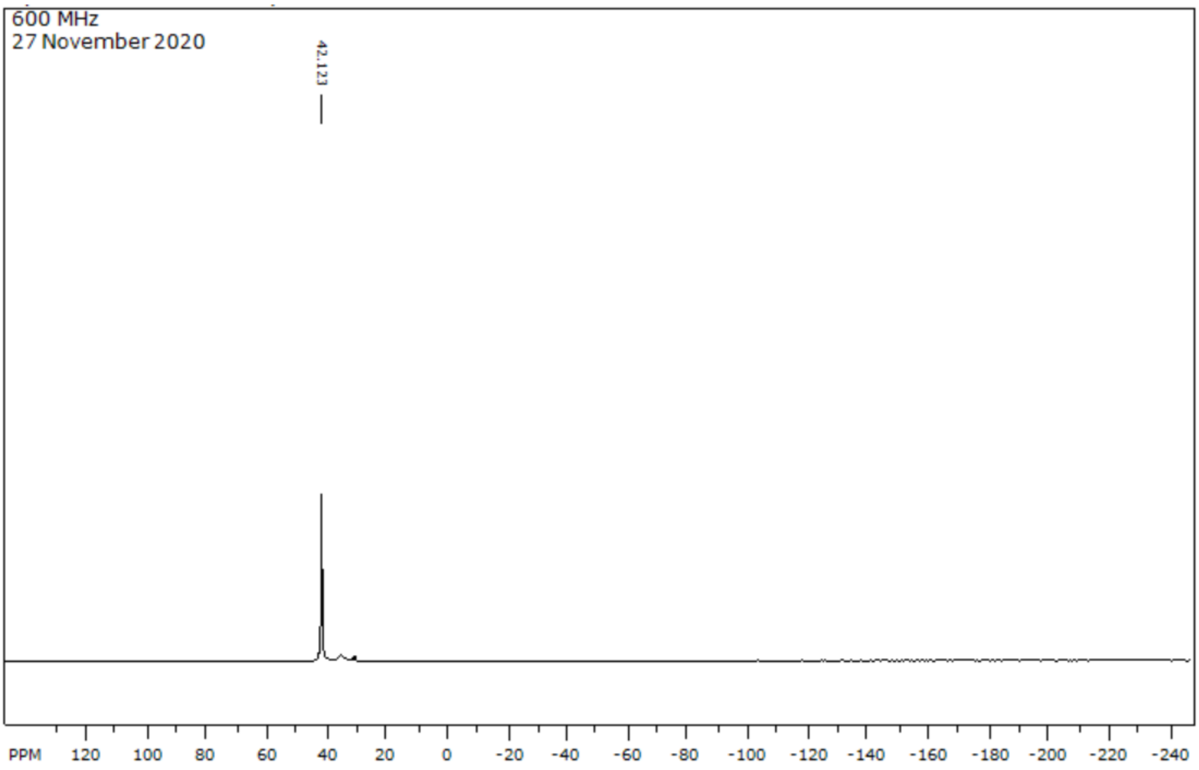

Figure S7. ^{31}P NMR for complex 4.

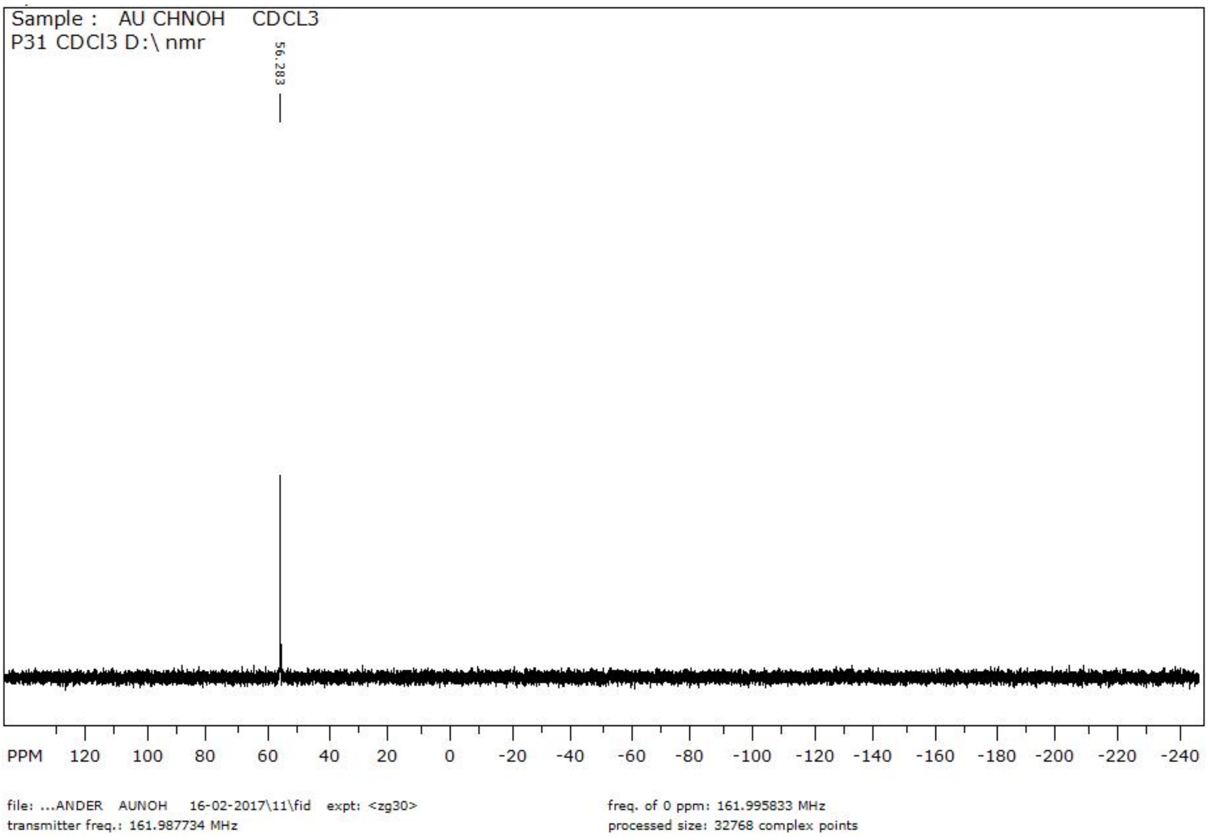

Figure S8. ^{31}P NMR for complex 5.
